# Chitosan/Graphene Oxide Nanocomposite Membranes as Adsorbents with Applications in Water Purification

**DOI:** 10.3390/ma13071687

**Published:** 2020-04-04

**Authors:** Alexa-Maria Croitoru, Anton Ficai, Denisa Ficai, Roxana Trusca, Georgiana Dolete, Ecaterina Andronescu, Stefan Claudiu Turculet

**Affiliations:** 1 Academy of Romanian Scientists, Spl. Independenței 54, 50085 Bucharest, Romania; croitoru.alexa@yahoo.com (A.-M.C.); ecaterina.andronescu@upb.ro (E.A.); 2 Faculty of Applied Chemistry and Materials Science, University Politehnica of Bucharest, Gh. Polizu St 1-7, 011061 Bucharest, Romania; denisaficai@yahoo.ro (D.F.); truscaroxana@yahoo.com (R.T.); dolete.georgiana@gmail.com (G.D.); 3 Faculty of Medicine, Carol Davila University of Medicine and Pharmacy, Eroii Sanitari St 8, 050474 Bucharest, Romania; turculet.claudiu@yahoo.com; 4 Emergency Hospital Floreasca Bucharest, Calea Floreasca St 8, 014461 Bucharest, Romania

**Keywords:** chitosan, graphene oxide, EDTA, lead, nanoadsorbent

## Abstract

The scope of this article is to develop composite membranes using chitosan (CS) and graphene oxide (GO) as adsorbents for the removal of inorganic pollutants such as heavy metal ions, particularly Pb^2+^, from aqueous solutions. GO was obtained by modified Hummers method and blended with CS solution. The introduction of ethylenediaminetetraacetic acid (EDTA) compound to CS/GO suspension lead to an increased adsorption capacity of CS/GO for the elimination of heavy metals by forming stable chelates with them. The synthesized membranes were examined by Fourier transform infrared spectroscopy (FTIR) and scanning electron microscopy (SEM), and the adsorption behaviour of Pb^2+^ from aqueous solutions using CS/EDTA/GO membranes was evaluated using inductively coupled plasma mass spectrometry (ICP-MS). The adsorption performance of Pb^2+^ ions was studied by monitoring the concentration of Pb^2+^ against the adsorption period at an initial content of the adsorbent. The maximum adsorption efficiency of Pb^2+^ metal ions reached 767 mg·g^−1^ for CS/EDTA/GO 0.1%, 889 mg·g^−1^ for CS/EDTA/GO 0.3%, 970 mg·g^−1^ for CS/EDTA, 853 mg·g^−1^ for CS and 1526 mg·g^−1^ for GO. These findings show promising potential for CS/EDTA/GO membranes as effective adsorbent materials for the removal of heavy metal ions in water.

## 1. Introduction

In the last several years, the presence of toxic pollutants (antibiotics, pesticides, heavy metals, etc.) in wastewater has become a concerning problem affecting the ecological environment and human health in the long term. The enhancement of heavy metal ion concentrations in potable water, surface water and ground water affect human and animal life [1]. Thus, the elimination of contaminants like inorganic metal ions from wastewater is very important, as they are encountered infertilizer, the production of different plastics, mining, metallurgy, electroplating, chemical manufacturing and so forth [2,3,4,5]. Heavy metals can have a dangerous impact on human health, even at low concentrations. Compared to organic contaminants, heavy metals (Cr^6+^, Pb^2+^, Hg^2+^, etc.) are imperishable and tend to accumulate in living organisms, causing toxic effects such as high blood pressure, fatigue, irritability, increased allergic reactions and autoimmune diseases [6]. At present, numerous techniques are used for the removal of metal ions, including reduction, co-precipitation, UV photolysis/photocatalysis, membrane filtration, ion exchange and adsorption. Of all these methods, adsorption is the most significant process because of its advantages, such as the relatively low cost and convenience of use [7,8]. Therefore, new strategies have been developed using nanoadsorbents based on nanotechnologies [9,10]. Numerous porous materials have been studied and developed for use as adsorbents (activated carbon, graphene oxide (GO), zeolite, CuO, ZnO, silica and polymeric adsorbent) for the removal of pollutants [11]. Lately, carbon-based materials (nanotubes, graphene oxides, etc.) have been shown to be promising materials for different applications, such as surface-enhanced Raman scattering and metamaterials [12], solar cells [13], and batteries and other energy storage devices [14,15], but they also have been studied as adsorbents in environmental applications for water purification [16]. Among carbon materials, graphene oxide presents a high adsorption capacity because of its particular properties: high specific surface area, rich oxygen functional sites with both hydrophilic and hydrophobic groups and good thermal and chemical stability. Thus, graphene oxide can be used in the decontamination of wastewater due to its adsorption properties, having the ability to remove one or more toxic compounds [17,18,19].

Chitosan (CS) has been considered a potential adsorbent material for the elimination of toxic pollutants due to its amino and hydroxyl groups [20], physico-chemical properties such as chemical stability, and high reactivity and selectivity toward pollutants and outstanding chelation behavior [21]. CS is non-toxic and biodegradable, and its production cost is low [11].

Ethylenediaminetetraacetic acid (EDTA) has been studied in the elimination of toxic metals because it forms stable chelates with metal ions [8].

Based on the literature data, scientists studied the removal of organic and inorganic contaminants using new materials based on GO [22,23]. In the past few years, GO and its derivatives have been studied for their adsorption properties for organic and inorganic contaminants in aqueous solutions [24,25,26]. Harijan et al. [27] investigated the effective removal of Cr^6+^ from water using a GO material functionalized with polyaniline (PANI-GO). The maximum adsorption capacity for PANI-GO was up to 192 mg·g^−1^ in 20 min where pH was 6.5 and temperature was 30 °C. With the decrease of pH and enhancement of the initial concentration of Cr^6+^, the adsorption capacity for Cr^6+^ increased. EDTA functionalized with GO was used as adsorbent material for the elimination of Pb^2+^. The best adsorption efficiency reached 479 ± 46 mg·g^−1^ in 20 min at a pH value of 6.8 [8].

These findings show that GO and its derivatives can be efficient adsorbents for the environment, especially concerning water treatment. This study was realized to examine the adsorption process and the potential applications of CS/EDTA/GO membranes in the elimination of Pb^2+^ ions from wastewater. The adsorption properties of the obtained materials were investigated by monitoring the concentration of Pb^2+^ against the adsorption period at an initial content of the adsorbent.

## 2. Materials and Methods

Low molecular weight chitosan (MW of 190 kDa and deacetylation degree of 75–85%), phosphorus pentoxide, potassium peroxydisulfate and lead(II) chloride 99.999% were acquired from Sigma–Aldrich (Steinheim, Germany). Graphite (natural powder), potassium permanganate (Lach–Ner, Neratovice, Czech Republic), glacial acetic acid (Chimreactiv, Bucharest, Romania), sulphuric acid 95%–97% and ethylenediaminetetraacetic acid (EDTA) were from Merck (Darmstadt, Germany). Hydrogen peroxide 35% and hydrochloric acid were from Silal Trading (Bucharest, Romania).

### 2.1. Fabrication of GO

**Scheme 1:**
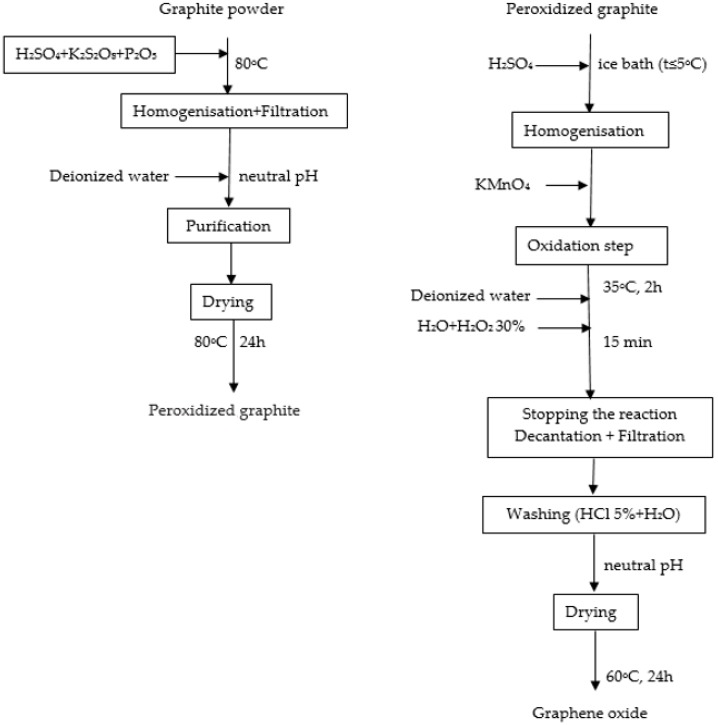
Synthesis of graphene oxide (GO) [29].

To synthesize GO, a modified Hummers method was used; the steps are presented in Scheme 1 [28,29]. Graphite powder (20 g) was blended with a mixed solution containing 60 mL H_2_SO_4_, 10 g P_2_O_5_ and 10 g K_2_S_2_O_8_ heated in advance to 80 °C. The solution was filtered after cooling and rewashed until the filtrate had no residual acid. The resulting material was dried at 80 °C for 24 h. The dried product (20 g) was added slowly to 460 mL of concentrated H_2_SO_4_ solution with a temperature below 5 °C. Sixty grams of KMnO_4_ was added slowly, and the suspension was stirred at 35 °C for 2 h until it became yellowish-brown. The solution was diluted with about 3 L of deionized water and stirred for 15 min and finally, to eliminate the excess of KMnO_4_, 50 mL of 30% H_2_O_2_ was added. The solution mixture was rewashed with 5% HCl and deionized water until the pH became neutral. The resulting product was dried in an oven for 24 h at 60 °C, and GO was obtained [29].

### 2.2. Preparation of CS/EDTA Composite Solution

A solution of chitosan (2%) was prepared in glacial acetic acid (90 wt%) and deionized water and mixed for 24 h at room temperature using a magnetic stirrer. Also, a solution of EDTA was prepared having a final concentration of 2% relative to chitosan. The two solutions were mixed and kept without stirring for 1 h at room temperature to allow the release of air bubbles after stirring.

### 2.3. Preparation of CS/EDTA/GO Films

**Scheme 2:**
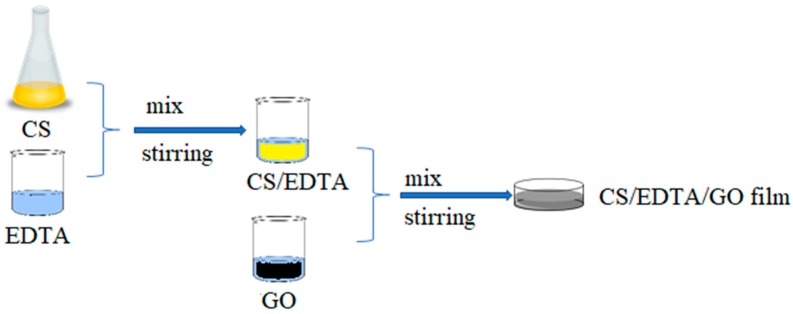
Schematic illustration of the preparation of chitosan (CS)/ ethylenediaminetetraacetic acid (EDTA)/GO films.

**Figure 1:**
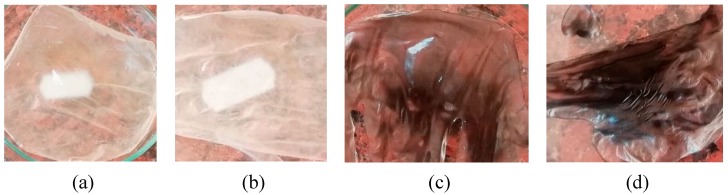
Digital photos of the samples: (**a**) chitosan (CS) film, (**b**) chitosan/ethylenediaminetetraacetic acid (CS/EDTA) film, (**c**) chitosan/ethylenediaminetetraacetic acid/graphene oxide (CS/EDTA/GO) film 0.1%, and (**d**) CS/EDTA/GO film 0.3%.

Known amounts of GO (0.01 and 0.03 g) were dispersed in 10 mL CS/EDTA and treated by mild sonication for 15 min until homogenous suspensions were formed. The obtained suspensions were then stirred for 1 h at room temperature and cast on two Petri glasses, forming a gel-like sheet with a thickness of approximately 1 mm. The composite membranes were dried in the oven for 24 h at 40 °C (Scheme 2). The CS and CS/EDTA films were prepared according to the same method. Figure 1 presents the obtained films.

The synthesized membranes were examined by IR spectroscopy using a Nicolet iS50 spectrophotometer (MA, USA) containing a deuterated triglycine sulfate (DTGS) detector that provided high sensitivity information. Measurements of the membranes were recorded from 4000 to 100 cm^−1^ with a resolution of 4 cm^−1^. All spectra were registered in Attenuated Total Reflectance (ATR) mode using a diamond crystal.

Morphological data were determined by scanning electron microscopy (SEM). The prepared films were characterized via a Quanta Inspect F50 electron microscope (Eindhoven, Nederlands) equipped with a field emission gun (FEG) having a resolution of 1.2 nm.

### 2.4. Adsorption Experiments

Lead concentration was determined using inductively coupled plasma mass spectrometry equipment (Agilent 8800 ICP-MS Triple Quadrupole, Agilent Technologies) (SC, USA). The measurements were performed for ^208^Pb isotope, and the quantification of the results was made by an external calibration method using a multi-element standard solution of 100 µg/mL for which the calibration curve points were prepared. The calibration curve for ^208^Pb isotope was linear in the range of 100–1300 µg/L with a correlation coefficient of R^2^ = 0.9985. The detection limit of the equipment for ^208^Pb was 0.01109 µg/L.

To determine the adsorption capacity, a solution of PbCl_2_ was obtained by dissolving 1.5 g PbCl_2_ (Pb^2+^ 1.1 g/L) in 1000 mL of deionized water. The adsorption experiments were realized by taking a fixed mass of dry film (20 mg) in 15 mL of metal solution. All samples were done in triplicate in order to avoid errors and to assess the homogeneity of the samples. As for GO, 20 mg of powder was added to 15 mL of metal solution. A quantity of 1 mL of each mixture was collected periodically (10, 20, 30, 45 and 60 min), and the final solutions were adjusted by serial dilution. Each sample was analyzed using ICP-MS to determine the adsorption capacity of each film. 

The following equation was used to calculate the amount of metal ions adsorbed on membranes [1]:

(1) $$q=\frac{\left(C_{0}-C_{e}\right)*V}{m}$$

where *q* is the adsorption capacity in mg·g^−1^, *C*_0_ and *C_e_* are the initial and equilibrium concentrations of Pb^2+^ in mg/L, *V* is the volume of the solution in L and *m* is the weight of the adsorbent in g.

## 3. Results

This paper presents the characterization of CS, CS/EDTA and CS/EDTA/GO membranes with different amounts of GO and their application as adsorbents for the elimination of Pb^2+^ ions from water.

**Figure 2:**
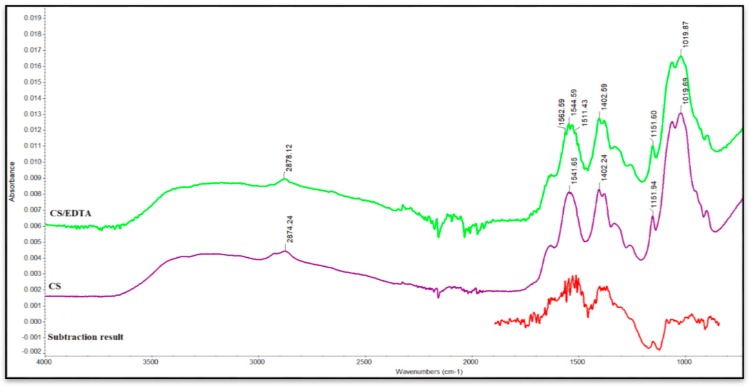
Fourier Transform Infrared Spectroscopy (FTIR) spectra for CS and CS/EDTA films.

Figure 2 shows FTIR spectra of CS and CS/EDTA films.

**Figure 3:**
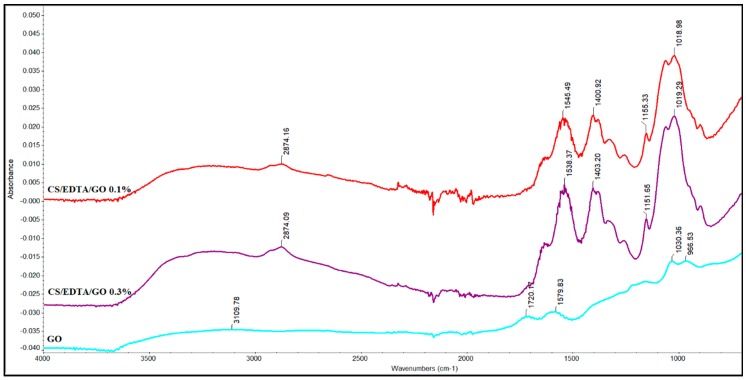
FTIR spectra for GO, CS/EDTA/GO 0.1% and CS/EDTA/GO 0.3%.

Figure 3 presents the FTIR spectra of GO and the as-prepared CS/EDTA/GO membranes at different GO concentrations.

**Figure 4:**
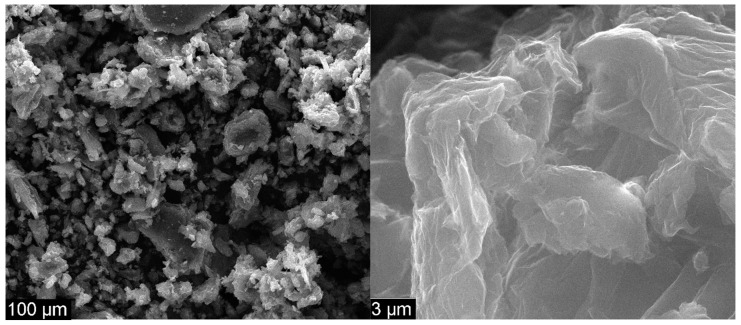
Scanning electron microscopy (SEM) images for GO at 1000 × (left) and 50 k× magnification (right).

Figure 4 presents the morphology of GO at 1000 × and 50 k× magnification.

**Figure 5:**
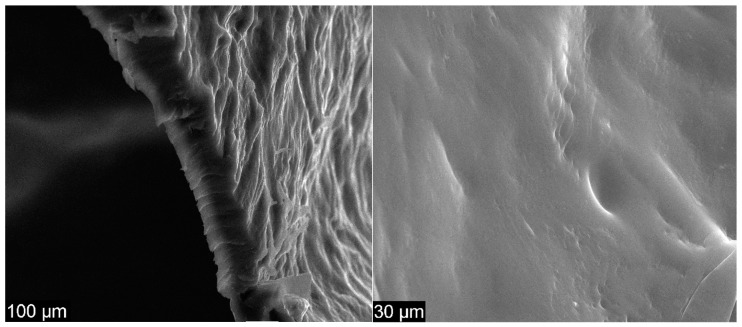
SEM images for CS at 1000 × (left) and 5000 × magnification (right)

Figure 5 presents the surface morphology of CS.

**Figure 6:**
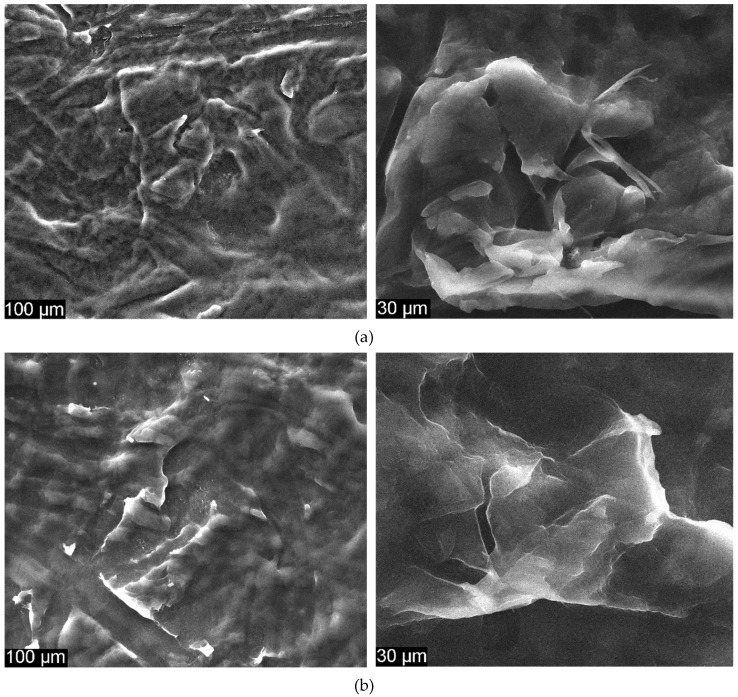

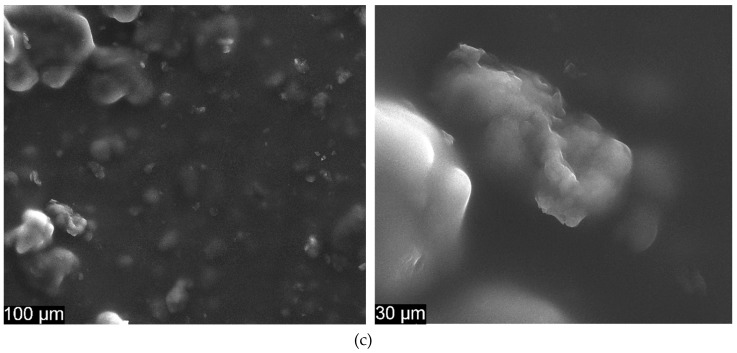
SEM images for (**a**) CS/EDTA, (**b**) CS/EDTA/GO 0.1% and (**c**) CS/EDTA/GO 0.3% at 1000 × (left) and 5000 × magnification (right).

Figure 6 presents the surface characteristics of CS/EDTA films and also shows SEM images of CS/EDTA and CS/EDTA/GO with different GO content.

Following the calculations, a maximum adsorption capacity was observed for each film of 767 mg·g^−1^ (CS/EDTA/GO, 0.1%), 889 mg·g^−1^ (CS/EDTA/GO 0.3%), 970 mg·g^−1^ (CS/EDTA), 853 mg·g^−1^ (CS) and 1526 mg·g^−1^ (GO).

**Figure 7:**
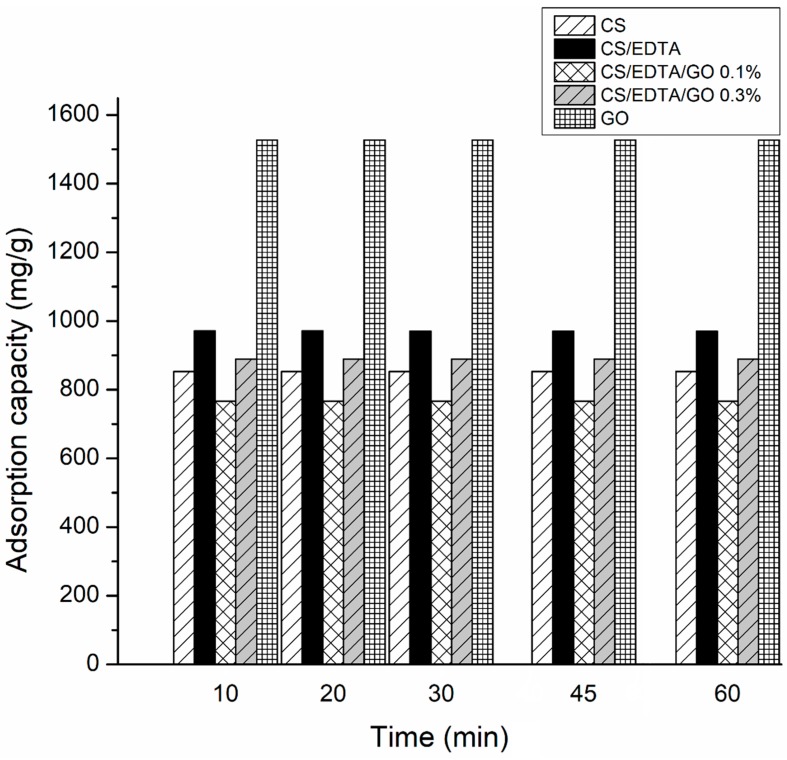
Sorption capacity of the membranes CS, CS/EDTA, CS/EDTA/GO 0.1%, CS/EDTA/GO 0.3% and GO sheets. * Standard Deviation (SD) = 1–3%.

Figure 7 shows the adsorption curves for GO and the prepared films.

## 4. Discussion

### 4.1. Fourier Transform Infrared Spectroscopy

Based on the IR spectrum of chitosan (Figure 2), it can be stated that the main characteristic peaks are clearly identified and are similar to those found in previous literature reports [30,31]. The wide absorption band between 3500 and 3000 cm^−1^ could be assigned to the –OH and –NH_2_ stretching vibrations of amino groups. The band between 2800 and 3000 cm^−1^ is attributed to the asymmetric stretching mode of –CH– groups of CS. The bands centered between 1450 and 1700 cm^−1^ are assigned to C=O stretching vibration (amide I) and N–H bending vibration (amide II). The absorption peak at 1402 cm^−1^ corresponds to C–N stretching vibration (amide III) in CS. The band recorded at 1019 cm^−1^ is attributed to C–O stretching vibrations [32,33]. The absorption bands were at 3430 and 1630 cm^−1^.

When compared with the original spectrum of CS, the FTIR spectrum of CS/EDTA (Figure 2) reveals several new absorption bands (1562 and 1511 cm^−1^, corresponding to the amide I and amide II groups). The absorption band around 3250 cm^−1^ corresponds to the stretching mode of -OH groups. The absorption band of the amide II group at 1541 cm^−1^ in CS shifts to 1544 cm^−1^ in the spectrum of the CS–EDTA film. Ionic bonds are formed when protonated amino groups of chitosan interact with carboxyl groups of EDTA, forming CS/EDTA suprastructures. By subtracting the spectrum of pure CS from the spectrum of the CS/EDTA nanocomposite, a visible widening of the peak around 1019 cm^−1^ can be seen, highlighting the interaction of CS with EDTA. The absorption band between 1500 and 1600 cm^−1^ corresponds to groups belonging to EDTA, and the band between 1100 and 1200 cm^−1^ is assigned to characteristic CS groups [34,35].

The FTIR spectra of GO (Figure 3) reveal specific bands of GO, similar to previous literature reports [33,36,37]. The peak from 1720 cm^−1^ is attributed to C=O stretching vibration present in the C=O and COOH of GO. The absorption band at 1579 cm^−1^ corresponds to C=C stretching vibration, and the bands at 1160 and 1030 cm^−1^ are attributed to COC and -COH stretching vibration groups. The broad peak at 3109 cm^−1^ is attributed to the associated -OH group from GO [33,36,37]. 

After the addition of GO, CS/EDTA/GO does not show important changes in FTIR peaks. However, a deformation in the vibration peak of amide I can be observed in conjunction with a shift in the band from 1545 to 1538 cm^−1^ due to the reaction of amino groups with epoxy groups, as stated by Cao et al. [38]. By subtracting the spectra of the two CS/EDTA/GO nanocomposites, an enhancement in the relative intensity of the peak from 1538 cm^−1^ can be observed due to the enhancement of GO concentration and the formation of hydrogen bonds between CS and GO, which demonstrates the interaction of GO with CS [39].

### 4.2. Scanning Electron Microscopy

The morphological structures of GO and chitosan-based membranes were determined by SEM at different magnifications, from 100 × to 50 k×. 

Figure 4 presents the structure of GO at 1000 × magnification as very thin agglomerated nanosheets. The waved and folded shape of GO is presented at 50 k× magnification, the results being similar to those found in previous studies [28,29,40,41]. The specific sheets of GO are not planar because GO has a high degree of functionalization. The width of GO was measured, and the results showed less than 20 µm per individual sheet; this finding is similar to the literature [42]. In Figure 5, the surface of CS shows a generally smooth and uniform morphology without perforations and with few banded structures, which are attributable to the tendency of chitosan to self-aggregate in solution. The clear surface indicates no presence of GO [32,33]. Furthermore, according to the SEM images realized in the transversal section, the thickness of the CS membrane, as well as the other membranes, was in the tens of microns. 

In Figure 6a, the illustrated CS/EDTA surface implies the unification of CS and EDTA in the composite material. Compared to pure CS, an increment in the density of the blended film can be seen, indicating a strong interaction between CS and EDTA [43].

A good dispersion of GO is also highlighted in CS/EDTA films (Figure 6b) without any aggregation, illustrating that CS/EDTA films blended with GO are compatible [44]. The inner structure of CS/EDTA/GO is much denser than the inner structure of CS, which leads to an increment in the mechanical strength of membranes [31,33]. Irregular wrapped zones assigned to the presence of GO platelets that can form during solvent evaporation (Figure 6b,c) [45] can be seen. Thus, amino groups in CS and EDTA groups can form chemical bonds with a GO surface [43].

### 4.3. Sorption Capacity of the Membranes

The functionalization of CS with EDTA and loading with GO sheets can lead to an increase in the adsorption capacity against heavy metals. The oxygen functional sites from GO and EDTA can make CS/EDTA/GO an ideal adsorbent material for the elimination of toxic inorganic pollutants such as Pb^2+^, Cu^2+^, Ni^2+^, Cd^2+^ and so forth. [8]. Based on the literature data, lead can form strong complexes with oxygen functional groups such as –COOH and –OH. The strong adsorption of Pb^2+^ can be attributed to the interactions between the cations of the metal ions and the oxygen groups of GO, especially –COOH and –OH [46].

Due to the strong and fast adsorption processes, these materials can be exploited in the elimination of Pb^2+^ ions from water solutions [8]. This study demonstrated that the adsorption capacity achieved an equilibrium state at ~20 min (Figure 7), the films having a maximum adsorption capacity of >99% when a standard solution of Pb^2+^ was used (15 mL, 100 ppm) for each 20 mg of adsorbent. After 20 min, a slight deterioration of the membranes was observed, and Pb^2+^ ions were marginally released in the solution. The obtained membranes revealed maximum adsorption capacities of 767 mg·g^−1^ (CS/EDTA/GO, 0.1%), 889 mg·g^−1^ (CS/EDTA/GO 0.3%), 970 mg·g^−1^ (CS/EDTA), 853 mg·g^−1^ (CS) and 1526 mg·g^−1^ (GO). For each membrane, three samples were analyzed, and the standard deviation value was between 1% and 3%. The results showed a higher adsorption capacity of CS/EDTA/GO 0.3% compared to that of CS/EDTA/GO 0.1%, which can be attributed to the increase in GO concentration. The lower performances observed in CS/EDTA/GO compared to CS/EDTA membranes can be attributed to the the chemical interaction between negative groups of CS, EDTA and GO such as –NH_2_, –OH and –COOH groups with metal ions that inhibit the adsorption of Pb^2+^ ions on the membranes. Nevertheless, the addition of GO to CS/EDTA film is of great interest due to the abundant oxygen functional sites, leading to an increase in adsorption capacity against heavy metals [47].

## 5. Conclusions

GO-based CS membranes functionalized with EDTA were successfully prepared using an aqueous solution of acetic acid as solvent. Adding EDTA to CS solution can help increase the adsorption process for Pb^2+^ ions. Hydroxyl and carboxyl groups from both GO and EDTA can make CS/EDTA/GO ideal adsorbents for the elimination of inorganic metal ions, such as Pb^2+^, Cu^2+^, Ni^2+^, Cd^2+^ and so forth, from wastewater. GO powder disperses very well in CS solution, creating strong hydrogen bonds between the –OH groups of CS and –OH groups of GO. The obtained films showed high affinity for Pb^2+^ ions in aqueous solutions with maximum adsorption values of 767 mg·g^−1^ (CS/EDTA/GO, 0.1%), 889 mg·g^−1^ (CS/EDTA/GO 0.3%), 970 mg·g^−1^ (CS/EDTA), 853 mg·g^−1^ (CS) and 1526 mg·g^−1^ (GO). It is important to mention that CS/EDTA/GO membranes had lower performances as compared to CS/EDTA, most probably because some of the –NH_2_, –OH and –COOH groups of the CS, EDTA and GO formed strong H bonds unavailable for binding Pb^2+^. Because GO is more expensive than other natural and carbon materials, it is important to fabricate a low-cost adsorbent material that has great adsorption capacity. Thus, the functionalization of CS with EDTA and GO can make CS/EDTA/GO an excellent adsorbent material for heavy metal removal. This work demonstrates that CS-based membranes have great adsorption properties in the removal of toxic metal ions from wastewater. Following these results, other promising applications of these membranes can be proposed, such as biomaterials or bioactive packaging materials for the food industry.
